# Editorial: Environmental exercise physiology towards global warming: challenges, applications and future trends

**DOI:** 10.3389/fspor.2023.1243587

**Published:** 2023-07-05

**Authors:** Hidenori Otani, Kazunobu Okazaki, Hiroshi Hasegawa

**Affiliations:** ^1^ Faculty of Health Care Sciences, Himeji Dokkyo University, Himeji, Japan; ^2^ Research Center for Urban Health and Sports, Osaka Metropolitan University, Osaka, Japan; ^3^ Graduate School of Humanities and Social Sciences, Hiroshima University, Hiroshima, Japan

**Keywords:** global warming, climate change, heat stress, physical activity, thermal strain

**Editorial on the Research Topic** Environmental exercise physiology towards global warming: challenges, applications and future trends

Global warming is advancing and unstoppable. At the same time as mitigating environmental heat stress, humans are required to adapt to climate change through physical activity (i.e., exercise and work). Therefore, the field of environmental exercise physiology, which specializes in these areas, must consider adaptation strategies to heat stress. Based on the scientific interest in this latter field, this research topic is dedicated to the findings of environmental exercise physiology towards heat stress. The seven papers; five original articles, one mini review and one brief research report, published on the present topic exhibited their aims with varied perspectives. We will briefly introduce each of these projects in the next section.

Wen et al. demonstrated the recent increased interest in studies on the influence of pre-cooling and intermittent mixed-cooling by a combination of external and internal cooling on the executive function following exercise. The study examined this influence by performing a treadmill running simulating a tennis match in hot and humid environments. The authors found that such cooling methods enhance executive functions following exercise and prolong exercise duration compared to no cooling. That finding would provide evidence concerning the effectiveness of practical cooling strategies prior to and during exercise-heat stress.

Hutchins et al. conducted a systematic scoping review to investigate the literature on women's response to cold water immersion as a treatment for exertional heat stroke. This review also aimed to clarify whether current guidelines have appropriately considered research investigating women. The authors detected that women may cool faster than men during cold water immersion following exercise because of a rapid cooling response. They concluded that the current guidelines for exertional heat stroke are predominantly informed by research on males but have been accepted as appropriate for women without validation. That observation would contribute to the literature on the validity of a useful cooling technique following heat stress exercise.

Jiang et al. conducted a systematic review and a meta-analysis to study the literature on the usefulness of various external cooling methods on various exercise performance in hot environments. The authors revealed that central cooling (face, neck, head and torso cooling) would be a more effective strategy for improving exercise performance with improvements in skin temperature and thermal sensation in the heat compared with peripheral cooling (four limbs cooling). That result would make a contribution as solid evidence of cooling benefits during exercise in the heat.

Naito et al. showed an increasing interest in research regarding the cooling power of the fan cooling garments. The study tested the impacts of the use of the fan cooling jacket on thermoregulatory responses during recovery following exercise in the heat outdoors. The authors unearthed that external cooling by this jacket use during post-exercise recovery in addition to internal cooling by cold water ingestion induces a faster rate of decrease in body temperature (tympanum and skin) compared to internal cooling by cold water ingestion alone. That evidence would usher new insights into cooling techniques for recovery from fatigue following outdoor exercise in hot conditions.

Tokizawa carried out the study regarding the effect of wearing a wet inner garment under a ventilation garment (i.e., fan cooling garment) on thermal strain during exercise in the heat. The author investigated this effect using a water-soaked inner T-shirt with a ventilation jacket during intermittent walking in young and older men. He found that the combination of these garments mitigates thermal strain and lowers whole-body sweat losses during moderate-intensity work in hot environments in both young and older men. That observation would provide new evidence for developing cooling methods during work-heat stress.

Iwahashi et al. examined the effects of cold water immersion of the hands and forearms during half-time on thermoregulatory responses and exercise performance by imitating intermittent athletic games in hot conditions. They found that such water immersion improves subsequent intermittent exercise performance and alleviates thermal and perceptual strain. That finding would lead to emerging trends as a practical cooling strategy between exercise bouts in hot environments in athletic settings.

Otani et al. studied the time-of-day effects of team training sessions in the gym without airflow and air conditioning on thermal strain in the summer heat. The authors observed that thermal strain is greater in the late afternoon from 4 p.m. than in the morning from 9 a.m. during 2.5 h badminton training sessions in the gym without airflow and air conditioning under these conditions. That result would help to the knowledge of preventing heat-related illnesses during exercise in the summer heat.

To conclude, this Research Topic demonstrates that the research on environmental exercise physiology continues to develop to challenge global warming. We hope that the papers presented in the present Research Topic make a contribution as an encouragement for new research ideas against global warming.

However, there are still many unsolved issues for global warming adaptation in terms of environmental exercise physiology. For instance, what combination of interventions (i.e., heat acclimatisation/acclimation, aerobic training, conditioning and training procedures, supplement consumption, etc.) is required to substantially increase tolerance to heat stress? What is the most effective combination of heat mitigation strategies (i.e., cooling, fluid ingestion, food intake, clothing, physical condition, etc.) to alleviate thermal strain under various environmental conditions (i.e., ambient temperature, humidity, wind speed, solar radiation, time-of-day, etc.)? It is expected that these issues will be clarified in future research projects to build up this research area.

Finally, we would like to thank the cooperation of Prof. Burtscher and Prof. Stapley, who helped us as the editor, and all reviewers.

